# Improved biological activity of a mutant endostatin containing a single amino-acid substitution

**DOI:** 10.1038/sj.bjc.6601745

**Published:** 2004-03-30

**Authors:** Y Yokoyama, S Ramakrishnan

**Affiliations:** 1 Department of Pharmacology, University of Minnesota, 6-120 Jackson Hall, 321 Church Street, S.E., Minneapolis, MN 55454, USA; 2 Obstetrics and Gynecology, University of Minnesota, Minneapolis, MN, USA; 3 Comprehensive Cancer Center, University of Minnesota, Minneapolis, MN, USA

**Keywords:** endostatin, point mutation, tumour angiogenesis, ovarian cancer, colon cancer

## Abstract

Human endostatin has an internal Asn-Gly-Arg (NGR) motif at position 126–128 following a proline at position 125. Asn-Gly-Arg-containing peptides have been shown to target tumour vasculature and inhibit aminopeptidase N activity. We previously compared the *in vitro* and *in vivo* biological activities of native endostatin and endostatin with a proline to alanine mutation (P125A-endostatin). P125A-endostatin exhibited greater inhibition of both endothelial cell proliferation and human ovarian cancer growth compared to native endostatin. Here we explore further the effects on biological activity of the P125A mutation, and show that aminopeptidase N is not involved. To determine whether the increased biological activity of the mutant was due to unmasking of downstream NGR-sequence, effect of endostatin on aminopeptidase N activity was investigated. Neither the native nor the P125A-endostatin inhibited aminopeptidase N. However, synthetic peptides consisting of the S118-T131 region of endostatin inhibited aminopeptidase N. These results suggest that the internal NGR site in native or mutant endostatin is not accessible to aminopeptidase N, and that this activity is not involved in the enhanced biological activity of the P125A form. P125A-endostatin bound to endothelial cells more efficiently than native endostatin and exhibited greater inhibition of not only proliferation but also migration of endothelial cells. P125A-endostatin also localised into tumour tissue to a higher degree than the native protein, and displayed greater inhibition of growth of colon cancer in athymic mice. Both proteins inhibited tumour cell-induced angiogenesis effectively. Real-time PCR analysis showed that both native and P125A-endostatin decreased expression of key proangiogenic growth factors. Vascular endothelial growth factor and angiopoietin 1 were downregulated more by the mutant. These studies suggest that the region around P125 can be modified to improve the biological activity of endostatin.

Endostatin is a proteolytic fragment of the noncollagenous domain of collagen type XVIII (O'Reilly *et al*, 1997), a component of the basement membrane. Endostatin inhibits growth factor-induced proliferation and migration of endothelial cells *in vitro* and angiogenesis *in vivo*. A number of independent studies have shown that endostatin treatment inhibits tumour growth by blocking angiogenesis (Boehm *et al*, 1997; O'Reilly *et al*, 1997; Dhanabal *et al*, 1999). Endostatin binds to at least two distinctive sets of molecules on the endothelial cell surface, *α*_5_*β*_1_/*α*_v_*β*_3_ integrin (Rehn *et al*, 2001; Wickstrom *et al*, 2002) and glycosyl-phosphatidylinositol (GPI) anchored heparin sulphate proteoglycan (HSPG), or glypican (Karumanchi *et al*, 2001). Glypican is believed to sequester endostatin and present it to the integrins, thereby forming a receptor-signalling complex. Binding to integrins is linked to phosphorylation of SH2 containing Shb adaptor protein, which is implicated in the apoptotic cascade (Dixelius *et al*, 2000). Such interactions can activate intracellular signalling leading to inhibition of endothelial cell proliferation and migration (Rehn *et al*, 2001; Shichiri and Hirata, 2001). A recent study showed that wnt-signalling pathways might also be modulated by endostatin (Hanai *et al*, 2002). In addition to these direct actions, endostatin has been shown to bind and inactivate metalloproteinases *in vitro* (Kim *et al*, 2000). These studies collectively imply that the mechanism of endostatin action is diverse and complex. Understanding the structure/function of endostatin therefore will help in improving its efficacy to inhibit tumour growth.

Human endostatin containing a point mutation at position 125 has been identified during expression cloning (Yokoyama and Ramakrishnan, 2002). Proline 125 is followed by a tripeptide, Asn-Gly-Arg (NGR), a sequence that is known to target endothelial cells (Pasqualini *et al*, 2000). In fact, chemical linkage of doxorubicin to NGR peptides inhibited tumour growth efficiently (Arap *et al*, 1998). Asn-Gly-Arg sequence has also been shown to bind and inhibit aminopeptidase N localised on vascular endothelial cells of tumours. The P125A-mutation is not a conservative change and would be expected to alter peptide folding around the mutation site. However, we were able to express P125A-endostatin in yeast in fully soluble form, and showed that it has similar gross secondary structures as native endostatin. P125A-endostatin inhibited *in vitro* endothelial cell proliferation and *in vivo* growth of ovarian cancer more effectively when compared to native endostatin (Calvo *et al*, 2002).

In this study, we have further explored the differences between native and P125A-endostatin. We report that the mutant protein binds more effectively to endothelial cells and is more effective in inhibiting not only endothelial cell proliferation but also migration *in vitro*. P125A-endostatin also displayed improved inhibition of colon cancer growth in athymic mice, and greater downregulation of human vascular endothelial growth factor (VEGF) and human angiopoietin 1 (Ang1) from tumours. Neither native nor mutant endostatin inhibited aminopeptidase N activity. These studies suggest that structural changes in endostatin can be used to improve the biological activity of human endostatin.

## MATERIALS AND METHODS

### Cell lines and culture conditions

Bovine adrenal gland capillary endothelial (BCE) cells were obtained from Clonetics Inc. (San Diego, CA, USA). Culture conditions of human umbilical vein endothelial cells (HUVEC) have been published previously (Ramakrishnan *et al*, 1996). Human colon carcinoma cell line, LS174T, was obtained from American Type Culture Collection (ATCC, Rockville, MD, USA). LS174T cells were cultured in RPMI-1640 (GIBCO BRL, Gaithersburg, MD, USA) supplemented with 10% FBS, 100 U ml^−1^ penicillin, 100 *μ*g ml^−1^ streptomycin and 2 mM L-glutamine.

### Cloning, expression and purification of mutant human endostatin

The following primers were used to amplify the C-terminal end of collagen type XVIII (183 amino acid residues) by RT–PCR:

*Up*: GGGGAATTCCACAGCCACCGCGACTTCCAG,
*Down*: GGGGCGGCCGCCTACTTGGAGGCAGTCATGAAGCT.

The PCR product was cloned into pPICZ-*α*A vector (Invitrogen, Carlsbad, CA, USA) and sequenced. Selected clones were electroporated into X-33 host strain of *Pichia pastoris* (Invitrogen). Previously published methods were followed for expression and purification of endostatin (Yokoyama *et al*, 2000).

### Structural analysis by circular dichroism

Circular dichroism (CD) studies of endostatin and P125A-endostatin were carried out in a JASCO J-710 spectropolarimeter. Protein samples were prepared in PBS at a concentration of 100 *μ*g ml^−1^. Path length of the cell was 0.1 cm. Circular dichroism spectra and molar ellipticity were obtained over the wavelength range of 195–260 nm.

### Cell attachment assay

The method of cell attachment assay described previously was used (Maeshima *et al*, 2000). A measure of 1 nmole well^−1^ of endostatin preparations or 0.2% gelatin were used to coat 96-well plates. Human umbilical vein endothelial cells were prelabelled with a vital, fluorescence dye, 5 *μ*M 5-(and-6)-carboxyfluorescein diacetate, succinimidyl ester (5(6)-CFDA) (Molecular Probes, Eugene, OR, USA) for 10 min at 37°C. Cells were added to wells at a density of 40 000 cells well^−1^. After 1 h incubation at 37°C, unbound cells were removed, and fraction of bound cells was determined by a fluorescence plate reader (Cyto Fluor II; PerSeptive Biosystems, Framigham, MA, USA) (excitation, 485 nm, emission, 530 nm).

### Aminopeptidase N activity

Aminopeptidase N activity was determined by the method described previously (Arap *et al*, 1998). Human umbilical vein endothelial cell lysate (10 *μg* protein) was used as a source of aminopeptidase N and incubated with 0.225 mg (0.9 *μ*mol) substrate in a reaction buffer in the presence and absence of inhibitors at 37°C for 2 h. Aminopeptidase N activity was detected by absorbance at 405 nm.

### Endothelial cell proliferation assay

Essentially, the method described previously was used to determine the effect of endostatin on BCE cell proliferation (O'Reilly *et al*, 1997) by 3-(4,5-dimethylthiazol-2yl)-2,5-diphenyl-2,4-tetrazolium bromide, MTT (Carmichael *et al*, 1987; Yoon *et al*, 1999).

### Endothelial cell migration assay

The migration of endothelial cells was determined by using Boyden chambers (Neuro Probe, Gaithersburg, MD, USA) as previously described (Dhanabal *et al*, 1999).

### Tumour localisation

LS174T cells were injected subcutaneously at right and left sides of the flanks of athymic nude mice. Tumour size reached about 500 mm^3^ on day 10. Tumour-bearing mice were randomised into two groups. Endostatin or P125A-endostatin was injected at a dose of 20 mg kg^−1^ subcutaneously. Tumour tissues and representative normal tissues were surgically removed after 19 h. This time point was chosen to minimise overwhelming serum levels from obscuring the tissue-bound endostatin. For comparison, serum samples were also collected from the mice. Tissues were snap frozen, and homogenised in RIPA buffer containing proteinase inhibitors (PBS, 1% NP40, 0.5% sodium deoxycholate, 0.1% SDS, 10 *μ*g ml^−1^ PMSF), maintained at 4°C for 45 min, and cleared by centrifugation. Human endostatin concentrations in serum and tissue lysates were measured using an enzyme-linked immunoassay (Cytimmune, College park, MD, USA) according to the manufacturer's instructions. Statistical significance was determined by Student's *t*-test.

### Matrigel plug assay

Matrigel plug assay was used to determine inhibition of tumour cell-induced angiogenesis *in vivo* (Zhang *et al*, 2000). LS174T cell suspension was mixed with matrigel, and injected into nude mice subcutaneously. Mice were treated with native endostatin or P125A-endostatin at a dose of 20 mg kg^−1^day^−1^ subcutaneously at a distant site near the neck. Control mice were treated with an equal volume of sterile PBS at similar schedule. Treatment was started just after the matrigel implantation and continued for 1 week. At 1 day after the last treatment, the matrigel was removed. A part of the matrigel was used to prepare cryostat sections and another part of the samples was used for Real-time PCR analysis. Frozen sections (10 *μ*m) of matrigel were stained with 1 : 50 dilution of an anti-CD31 monoclonal antibody conjugated to phycoerythrin (MEC 13.3, BD PharMingen, San Diego, CA, USA) and analysed for vessel density (Wild *et al*, 2000). Serial frozen sections were used to localise VEGF by indirect immunoflourescence method using a polyclonal antiserum made against recombinant human VEGF_165_ (Olson *et al*, 1996). Another part of the matrigel samples were fixed in 10% neutral buffered formalin and processed for haematoxylin and eosin (H&E) staining.

### Quantitative real-time PCR analysis

Total RNA was extracted from frozen matrigel samples using the RNeasy Mini kit (Qiagen, Valencia, CA, USA) according to the manufacturer's protocol. Reverse transcription was performed with the SuperScript II kit (Invitrogen) using 1 *μ*g of total RNA. Real-time PCR was carried out by using SYBR Green PCR Master Mix (Applied Biosystems, Foster City, CA, USA) in an ABI PRISM 7700 sequence detection system (Applied Biosystems) (TaqMan). Primer sequences described by Gerber *et al* (2000) were used (Table 1

**Table 1:** Primer sequences used for real-time PCR

**Probe name**	**Forward primer**	**Reverse primer**
Human VEGF	AATGACGAGGGCGTGGAGT	TTGATCCGCATAATCTGCATG
Human bFGF	TGAATCACTAACTGACTGAAAATTGA	GAAGGGTCTCCCGCATACT
Human Ang 1	CCTTCCAGCAATAAGTGGTAGTT	CAAACGGCTCCAGATTCA
Human IL-8	TTTAGCATAGCTGGACATTAAAGAG	GCAAATATGCTTAGGCTTTAACC
Human GAPDH	CCACCCATGGCAAATTCCATGGCA	TCTACACGGCAGGTCAGGTCCACC
Mouse flt-1	GTCGGCTGCAGTGTGTAAGT	TGCTGTTCTCATCCGTTTCT
Mouse flk-1	TGTCAAGTGGCGGTAAAGG	CACAAAGCTAAAATACTGAGGACTTG
Mouse tie-2	CGGCCAGGTACATAGGAGGAA	CCCCCACTTCTGAGCTTCAC
Mouse endoglin	GCAGGCAAGAACTCAGACAT	AGCTCCCTCAGCTTCTGTTT
Mouse GAPDH	ATGTTCCAGTATGACTCCACTCACG	GAAGACACCAGTAGACTCCACGACA

) to amplify proangiogenic factors related messages from the human tumour cells and relevant target receptors on endothelial cells of mouse origin. Following conditions were used for PCR: 50°C for 2 min, 95°C for 10 min, and 40 cycles at 95°C for 0.15 min, 60°C for 1 min. Negative controls included omission of the template. SYBR Green dye intercalation into the minor groove of double-stranded DNA reaches an emission maximum at 530 nm. Relative RNA equivalents for each sample were calculated by either comparing to human or mouse GAPDH levels. Five to six samples per group were run in duplicate. Statistical analysis was performed by Student's *t*-test.

### Preparation of alginate beads encapsulated endostatins and tumour growth inhibition studies

Alginic acid extracted from *Macrocystis pyrifera* was purchased from Sigma Chemicals, St. Louis, MO. A measure of 4% (w/v) of alginic acid in water was sterilised by autoclaving. Endostatin preparations or PBS (control) made in 1.5% alginic acid were dropped gently into 0.1 M CaCl_2_ solution using a fine needle under aseptic conditions. Beads were kept at 4°C overnight and were washed with sterile water before the subcutaneous implantation into tumour-bearing mice. Entrapment efficiency was calculated by determining the amount of protein remaining outside the beads from the total protein using the BCA protein assay kit (Pierce, Rockford, IL, USA). Logarithmically growing LS174T cells were harvested by trypsinisation and suspended in serum-free medium at a density of 1 × 10^7^ cells ml^−1^. A measure of 100 *μ*l of the single-cell suspension was then subcutaneously injected into the flanks of female athymic mice (6–8 weeks old). When the tumours became visible (3 days after inoculation), mice were randomised into groups and treated with endostatin-encapsulated alginate beads (five animals per group). Endostatins were implanted at a distant site (about 2 cm away) at a dose of ∼20 mg kg^−1^ once a week. Tumour growth was monitored by periodic caliper measurements. Tumour volume was calculated by the following formula: tumour volume (mm^3^)=(*a* × *b*^2^)/2, where ‘*a*’=length in mm, ‘*b*’=width in mm. Statistical significance between control and treated groups was determined by Student's *t*-test.

## RESULTS

### Preparation and structural analysis of P125A endostatin

Native human endostatin and a mutant with a proline to alanine substitution at position 125 were cloned and expressed in *P. pastoris*. The P125A mutation did not change the binding of endostatin to heparin. Like native human endostatin, P125A-endostatin bound to a heparin–ceramic column and eluted at around 300 mM NaCl concentration, indicating similar binding strength to heparin (data not included).

**Figure 1:**
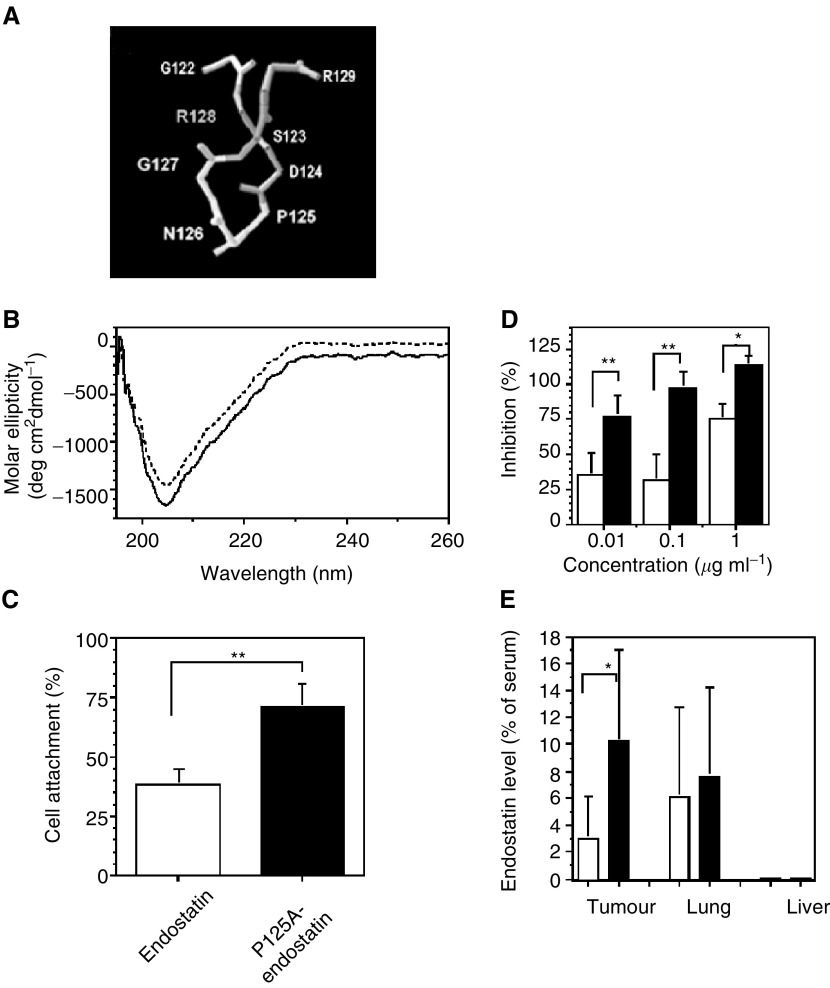
Characterisation of P125A–endostatin–increased binding and biological activity. (**A**) Structural details surrounding the mutation site are shown. Swiss PDB Viewer programme was used to generate the figure based on the structural information published by Ding *et al* (1998). P125 is followed by N126, G127, and R128. (**B**) Circular dichroism spectroscopy of endostatin (- - -) and P125A-endostatin (—). (**C**) Cell attachment assay. Single-cell suspension of HUVEC, prelabelled with 5(6)-CFDA, was added into triplicate wells coated with either endostatin or P125A-endostatin at a concentration of 1 nmol well^−1^. Wells coated with 0.2% gelatin were used as maximum attachment (100%). Bound cells were quantified by a fluorescence plate reader. Values represent mean of two independent experiments. Data are expressed as a mean (columns)±s.d. (bars). Statistical significance was determined by Student's *t*-test. ^**^P<0.01. (**D**) Effect of endostatin (open bars) and P125A-endostatin (closed bars) on endothelial cell (HUVEC) migration. Basic FGF (25 ng ml^−1^) was used to induce migration of endothelial cells. Data are expressed as a mean (columns)±s.e. (bars). Statistical significance was determined using Student's *t*-test. ^*^P<0.05, ^**^P<0.01. (**E**) Tumour Localisation. Human colon carcinoma cells (LS174T) were injected s.c. into female athymic nude mice. When the tumour size was around 500 mm^3^ (10 days after inoculation), Endostatin (open bars) or P125A-endostatin (closed bars) was injected at a dose of 20 mg kg^−1^ subcutaneously. Endostatin levels were determined by ELISA. Endostatin levels are expressed as a percent of serum levels.

An NGR-sequence capable of targeting endothelial cells is located immediately following the P125A-mutation site. Conformational features surrounding the mutation site are shown in Figure 1A

. Crystallographic data published by Ding *et al* (1998) show that P125 is located in a loop flanked by two *β*-sheets. In order to determine whether the mutant protein was folded properly, gross secondary structural analysis of the P125A mutant was compared to the native protein made in the same expression system. The CD spectra of native and P125A human endostatin showed identical profiles, indicating that the two proteins have similar gross secondary structures (Figure 1B).

### P125A mutation-enhanced endothelial cell binding

The biological characteristics of P125A-endostatin were compared to those of the native protein in a number of assays. As a first step, the ability to bind endothelial cells was assessed using cell-attachment assays. Gelatin (0.2%)-coated wells were used as a control, with the number of cells (HUVEC) attached to gelatin-coated wells taken as 100% to calculate relative binding. By this standard, 38.5% of HUVEC attached to endostatin-coated wells. Under similar conditions, a significantly higher number of HUVEC (71%) bound to wells coated with P125A-endostatin (Figure 1C) (*P*=0.005). The observed differences in cell attachment are not due to variation in coating efficiencies, which were determined by ELISA method.

### Inhibition of endothelial cell migration

A possible consequence of increased binding is increased biological activity. Previously, we showed that P125A-endostatin inhibited endothelial cell proliferation more efficiently than native endostatin did (Calvo *et al*, 2002). Endothelial cell migration assay is a more sensitive parameter to assess the biological activity of endostatin. Therefore, bFGF-induced migration of endothelial cells was determined in the presence of native and P125A-endostatin. Similar to the proliferation assays, this mutant endostatin was more effective than native protein in inhibiting cell migration (Figure 1D). At all three concentrations tested, P125A-endostatin inhibited endothelial cell migration more efficiently than native endostatin.

### Tumour localisation is improved by P125A-mutation

To assess whether the improved endothelial cell binding *in vitro* can translate into enhanced tumour homing *in vivo*, tumour localisation studies were performed. Endostatin and P125A-endostatin were injected subcutaneously into human colon cancer-bearing athymic mice. Tumour, lung, liver and serum samples were collected. Relative levels of endostatin in the tissues are shown in Figure 1E. Native endostatin accumulated in the tumour tissues at a level of 3.0% when compared to serum levels. P125A-endostatin, on the other hand, was found at a more than three-fold higher concentration in the tumour tissue (10.22% compared to serum levels). This difference was statistically significant (*P*=0.03). While the liver showed negligible amounts of endostatins, lung tissues had significant accumulation of both native and mutant endostatin. However, lung tissues did not show any statistical difference between native and P125A-endostatin accumulation.

### Effect of endostatin and P125A-endostatin on aminopeptidase N activity

As the mutation site is immediately followed by NGR motif, we next tested whether the mutant endostatin has aminopeptidase N inhibitory activity. Cellular extracts of aminopeptidase N enzyme were prepared from HUVEC. These cells express 8–10 times higher levels of aminopeptidase N compared to cancer cells such as B16F10 (mouse melanoma), U937 (human monocytic leukaemia cell line), MA148 (human ovarian cancer cell line) and LS174T (human colon carcinoma cell line). Confluent HUVEC lysate showed about three times higher activity than proliferating HUVEC lysate (data not shown).

**Figure 2:**
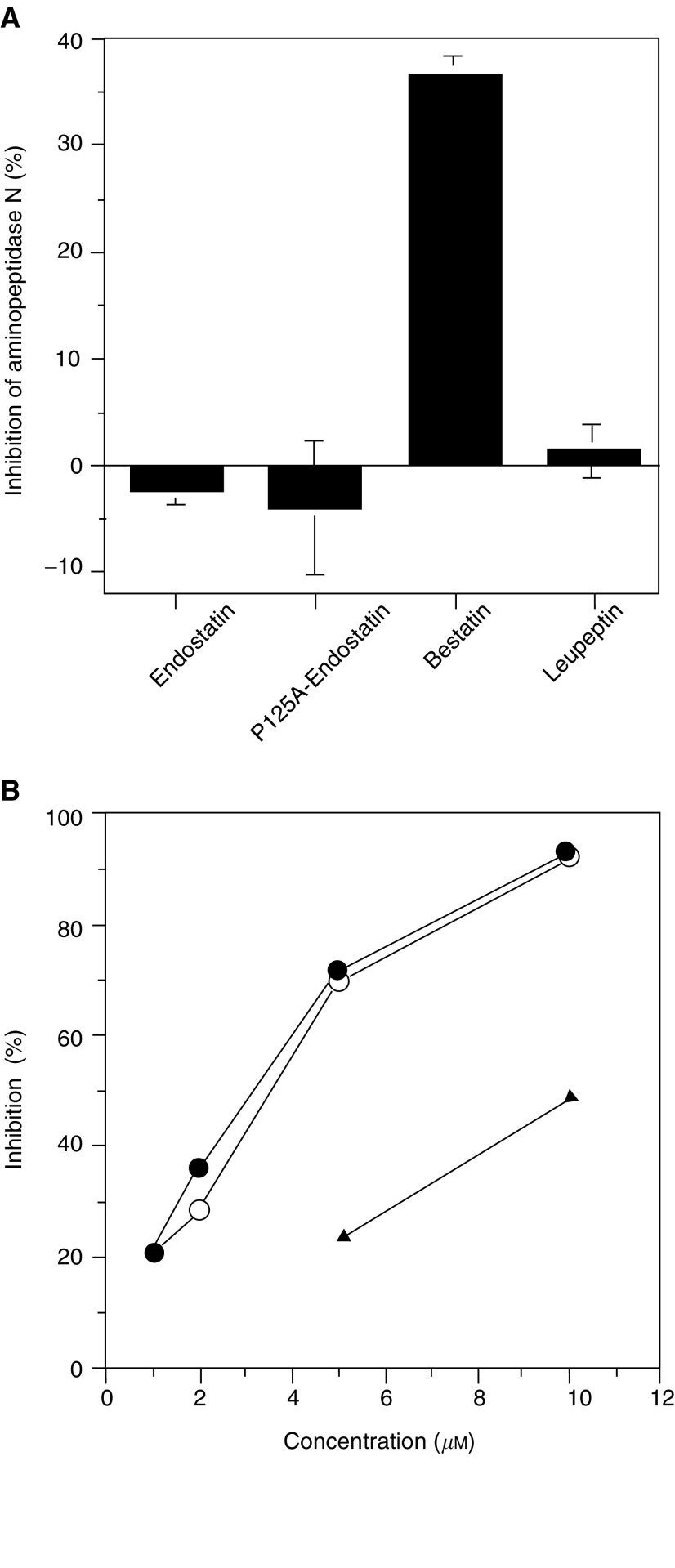
Effect of endostatin and synthetic peptides on aminopeptidase N activity. (**A**) Aminopeptidase N was extracted from HUVEC cultures. Bestatin was used as a positive control, and leupeptin treatment served as a negative control. Values represent mean of two independent experiments. (**B**) Two polypeptides containing 14 amino-acid residues (S118–T131) spanning the mutation site P125 were synthesised. One of the peptides had the native sequence and the other contained P to A substitution. Both peptides included the NGR motif. •, SR1 peptide (P125A); ○, SR2 peptide (native sequence); ▴, bestatin.

The data in Figure 2A

show the effect of endostatin and its mutant on aminopeptidase N activity. As a positive control, the same concentration of bestatin, a known inhibitor of aminopeptidase N, was included. Bestatin inhibited 35% of the enzymatic activity under the experimental conditions used. Leupeptin, a negative control, did not inhibit the enzyme. Interestingly, neither the native nor the P125A mutant showed any inhibition of aminopeptidase N activity even at 5 *μ*M concentration.

In a separate experiment, we tested whether synthetic peptides consisting of the amino acid sequence flanking the normal or mutated position 125 (S118-T131) could inhibit aminopeptidase N (Figure 2B). Interestingly, both peptides (native and mutant) inhibited aminopeptidase N activity more effectively than bestatin. At a concentration of 5 *μ*M, the mutant and native peptide inhibited aminopeptidase N by 71.2 and 69.2%, respectively. These studies suggest that the NGR motif in intact endostatin molecules constructs may not be accessible to interact with aminopeptidase N.

To confirm our findings, we characterised the interaction between endostatins and aminopeptidase N in an immunoabsorption assay using antibodies to human aminopeptidase N enzyme. Both synthetic peptides (A125 and P125) corresponding to the region S118-T131 were capable of binding to aminopeptidase N, to similar levels. However, intact proteins (native and P125A-endostatin) did not show any detectable binding to aminopeptidase N (data not included). These results further confirm that the NGR motif in endostatin is not accessible for binding to aminopeptidase N.

### Antiangiogenic activity of P125A-endostatin

**Figure 3:**
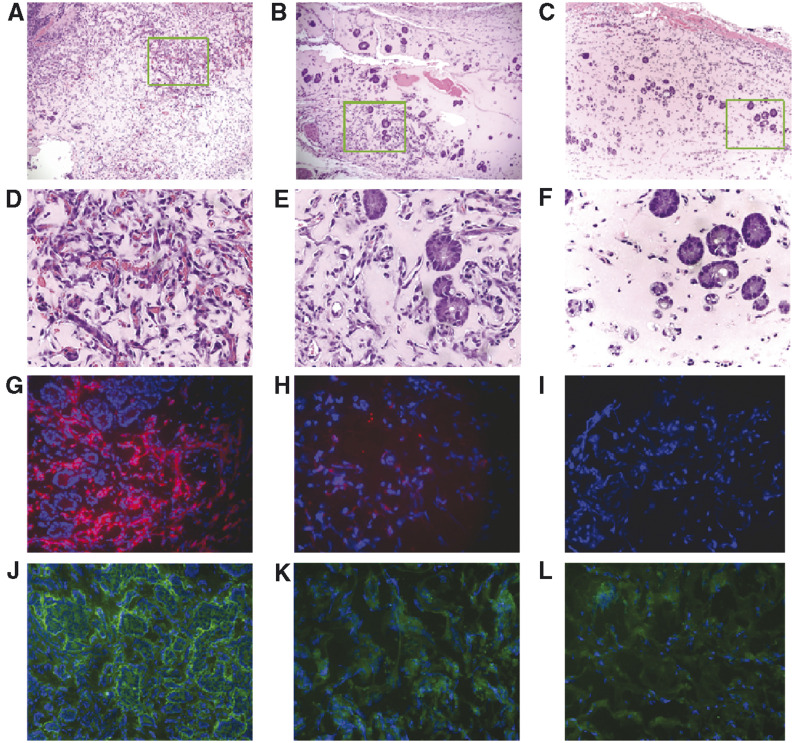
Histological analysis of matrigel plugs. Matrigel plugs containing LS174 human colon cancer cell line were used to determine the effect of endostatin and P125A-endostatin on angiogenesis. ((**A**)–(**F**)) Show H&E staining; ((**A**)–(**C**)) × 100 magnification, ((**D**)–(**F**)) × 400 magnification, Green squares in ((**A**)–(**C**)) indicate the area of the images in × 400 magnification of ((**D**)–(**F**)). ((**G**)–(**I**)) Show vessel staining with anti-CD31 antibody (red) and nuclei staining with DAPI (blue) × 200 magnification; ((**J**)–(**L**)) VEGF expression detected by indirect immunofluorescence using FITC-conjugated antibodies (green) with DAPI (blue). ((**A**), (**D**), (**G**), (**J**)) control; ((**B**), (**E**), (**H**), (**K**)) native endostatin-treated matrigel sections; ((**C**), (**F**), (**I**), (**L**)) P125A-endostatin-treated matrigel sections.

**Figure 4:**
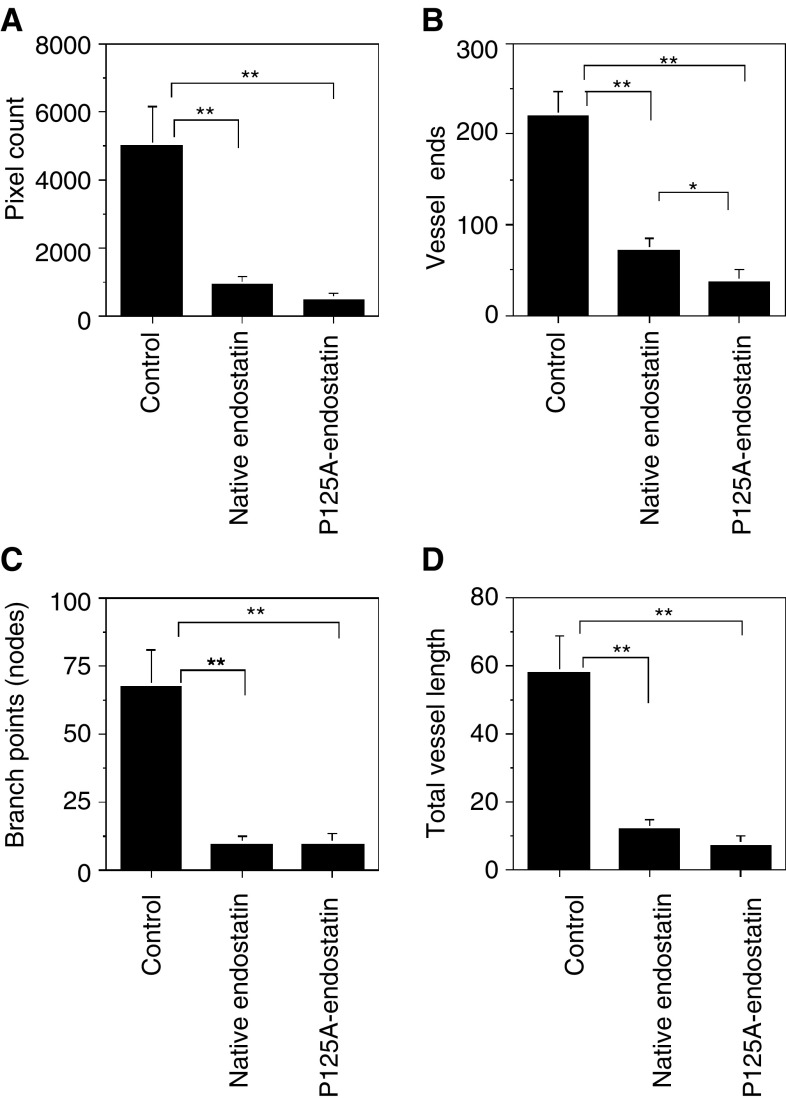
Inhibition of angiogenesis by native and mutant endostatin. A total of 7–10 frames of matrigel sections stained with anti-mouse CD31-PE were captured per sample and then analysed for microvessel density. (**A**) pixel density; (**B**) blood vessel ends (Ends); (**C**) branch points (nodes); (**D**) vessel length. Statistical significance was determined using Student's *t*-test. ^**^, *P*<0.01; ^*^, *P*<0.05. The error bars indicate s.e.

Next, we determined the ability of endostatin and P125A-endostatin to inhibit human colon cancer cell-induced angiogenesis *in vivo* using matrigel plug assays. Both endostatin and P125A-endostatin inhibited angiogenesis stimulated by LS174T colon carcinoma cells (Figure 3

). Histological investigation of matrigels from control and treated (native or mutant endostatin) animals showed higher tumour cell density interspersed with well-developed blood vessels in control matrigels when compared to endostatin-treated groups. Endostatin-treated groups showed some of the tumour cells organised into islands. (Figure 3B, C, E, and F) when compared to control group treated with PBS (Figure 3A and D). Anti-CD31 staining of frozen sections showed quantitative difference between control and treated groups (Figure 4

).

The overall indicators of angiogenesis, such as microvessel density (MVD), number of blood vessel ends (Ends), nodes (branch points), and length, showed that both endostatin and P125A-endostatin treatment significantly inhibited angiogenesis *in vivo* (Figure 3H, I and Figure 4). Furthermore, endostatin treatment seemed to alter angiogenic growth factor expression in the tumour cell microenvironment. Cryostat sections of matrigels showed reduced amounts of VEGF in immunofluorescence studies using a polyclonal antibody made against human VEGF_165_ (Figure 3K and L). Reduced VEGF levels may be a reflection of reduced number of tumour cells in the matrigel following endostatin treatment or due to an indirect effect of endostatin-mediated changes in the microenvironment.

### Downregulation of angiogenic factors and receptors by native and P125A-endostatin

Previously, we showed that the mammary gland of P125A-endostatin-treated C3(1)/SV40 transgenic mice exhibited decreased mRNA levels of VEGF, angiopoietin-2, flk-1, flt-1, tie-1 and cadherin-5 when compared to PBS-treated control. In the present study, a different model system was used to determine selective changes in tumour-induced neovascularisation. Matrigel plugs do not contain any other host cells or vasculature at the beginning of the experiment. Therefore, this model system is good to assess changes in tumour cell microenvironment following antiangiogenic therapy. To determine whether endostatin treatment altered RNA levels of proangiogenic factors from tumour and host receptors in newly formed blood vessels, real-time PCR was performed using total RNA isolated from the matrigel samples. Proangiogenic factors were detected by human gene-specific primers, and receptors were detected by mouse gene-specific primers (Table 1).

**Figure 5:**
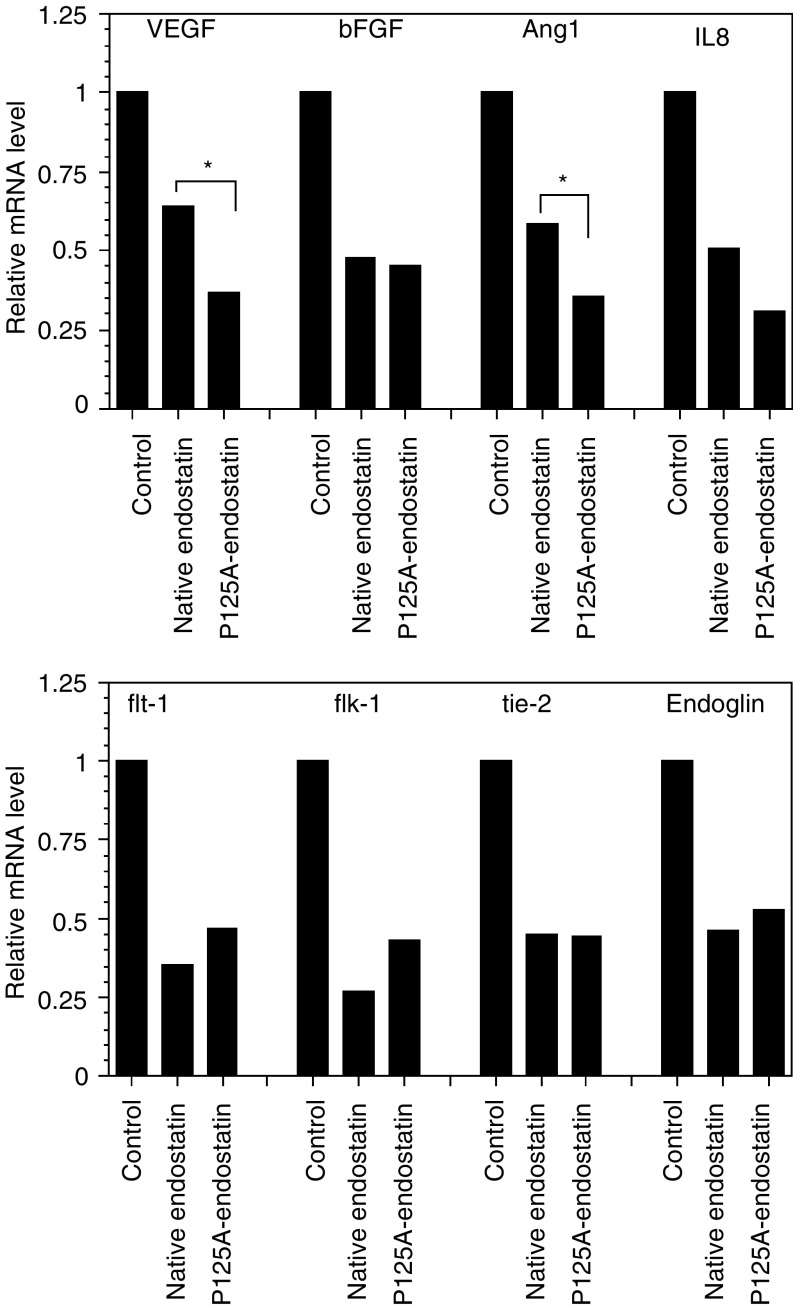
Downregulation of proangiogenic growth factors and receptors by endostatin treatment. Real-time PCR data were normalised by mRNA level to GAPDH. Data show relative mRNA levels. Messenger RNA of human proangiogenic factors (VEGF, bFGF, Ang1 and IL8) and mouse receptors (flt-1, flk-1, tie-2 and Endoglin) in Matrigel plugs were downregulated by endostatin and P125A-endostatin treatment.

Results shown in Figure 5

demonstrate that mRNA levels of major proangiogenic growth factors (tumour cell derived) and receptors expressed on host endothelial cells were downregulated by endostatin or P125A-endostatin treatment. Moreover, VEGF and Ang1 expression were significantly decreased by P125A-endostatin when compared to native endostatin. Angiopoietin 2 transcript was not detected in any of the samples analysed. Basic FGF-related transcript levels were equally decreased by both the native and mutant endostatin.

Target receptors for the tumour-derived angiogenic factors are located on the host vascular endothelial cells. Therefore, mouse-specific primers were used to study the levels of receptor molecules for VEGF and Ang1. These studies showed that both native and P125A-endostatin decreased mRNA levels of flt-1, flk-1, tie-2 and endoglin, a coreceptor for TGF-beta. Although native endostatin showed a slightly better effect when compared to the mutant protein in decreasing flk-1 and flt-1, the differences were not statistically significant.

### Inhibition of tumour growth by endostatin and P125A-endostatin

**Figure 6:**
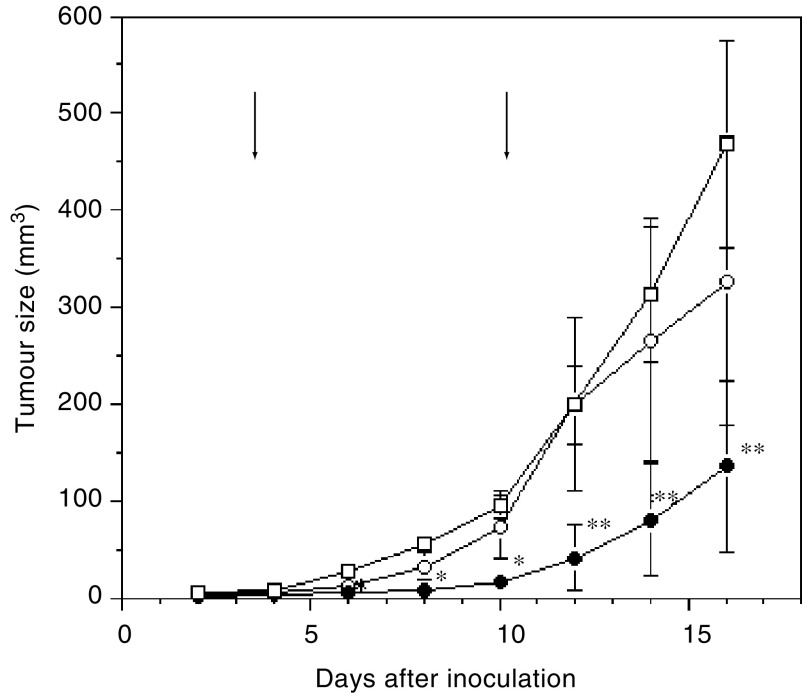
Improved inhibition of tumour growth by P125A-Endostatin. Human colon carcinoma cell line, LS174T was injected s.c. into female athymic mice. After tumours reached a palpable size, mice were treated with endostatin and P125A-endostatin encapsulated in alginate beads (s.c.) at a dose of 20 mg kg^−1^week^−1^. Endostatins were administered two times 1 week apart to colon cancer-bearing mice. Arrows denote time points at which microencapsulated endostatins were administered. Two endostatin treatments were given 1 week apart: □, alginate bead control (PBS); ○, endostatin; •, P125A-endostatin. The mean tumour volume of control and treated groups are shown. Statistical significance was determined using Student's *t*-test. ^*^P<0.05, ^**^P<0.01. The error bars indicate s.e.

Microencapsulated endostatin is more effective than bolus administration. In our previous studies, we showed that alginate beads of P125A-endostatin was more effective in inhibiting MA148 ovarian cancer growth when compared to the native protein given under similar condition (Calvo *et al*, 2002). These results were confirmed in a human colon cancer model system (Figure 6

). LS174T colon cancer cell line, which was used in the previous matrigel plug assay, grows aggressively in athymic mice and visible tumours can be seen as early as 3 days after subcutaneous injection. Endostatins encapsulated in alginate beads were given twice on days 3 and 10 at a dose of 20 mg kg^−1^. Although native endostatin exhibited only a marginal inhibition of LS174T tumour growth, P125A-endostatin treatment resulted in significantly enhanced antitumour activity. At the end of the experiment, native endostatin showed 30% inhibition of tumour growth, but P125A-endostatin decreased tumour size by 75% when compared to PBS-treated control animals. These studies confirm that P125A-endostatin inhibits tumour growth more effectively than the native protein.

## DISCUSSION

Human endostatin is a proteolytic fragment of collagen type XVIII (O'Reilly *et al*, 1997) and is generated *in situ* by elastase (Wen *et al*, 1999) and in the corneal epithelial cells by the action of matrilysin, MMP-7 (Lin *et al*, 2001). The *α*-1 chain of collagen XVIII is characterised by 10 domains of typical, triple-helical collagenous repeats separated by short noncollagenous regions (Oh *et al*, 1994). The carboxyl terminus 315 or 313 residues (mouse and human, respectively) are noncollagenous and form the NCI domain. Proteolytic processing of this domain results in the release of the C-terminal 183 or 181 residues of the NCI domain, endostatin. Collagen XVIII and its three splice variants are expressed in a tissue-specific manner and localised to the perivascular basement membrane. Endostatin-like sequence is also found at the C-terminal end of *α*-1 chain of collagen type XV (Ramchandran *et al*, 1999). Protein fragments from the NC1 domains of *α*-1 chain (arrestin), *α*-2 chain (canstatin) and *α*-3 chain (tumstatin) of collagen type IV are also effective in inhibiting angiogenesis and tumour growth (Kamphaus *et al*, 2000).

Recombinant mouse and human endostatins have been cloned and expressed (Dhanabal *et al*, 1999). In a number of model systems, endostatin treatment either inhibited or regressed experimental tumours (O'Reilly *et al*, 1997). In some studies, only moderate inhibition was observed (Dhanabal *et al*, 1999; Yokoyama *et al*, 2000). Understanding the basis for these discrepancies may help in the successful clinical development of endostatins. Alternatively, structure/function studies can be used to generate more potent angiogenic inhibitors. Proline 125 is located in a *β*-hairpin loop between the *β* sheets K and L of human endostatin. An endothelial cell homing motif, NGR, is located immediately following this mutation site in human endostatin. In mouse endostatin, however, the SGR sequence is seen in place of NGR. The NGR motif was originally identified while mining for sequences capable of homing to tumour vasculature using Phage display libraries (Pasqualini *et al*, 2000). In spite of the nonconservative substitution of a proline to alanine, the mutant protein was expressed in soluble form and was biologically active. The CD spectrum showed that P125A-mutation did not cause major structural change. Asn-Gly-Arg-containing peptides are known to inhibit endothelial cell membrane-associated aminopeptidase N activity. However, neither the native nor the mutant protein showed any detectable inhibition of aminopeptidase N. Although synthetic peptides corresponding to this region showed potent inhibition of aminopeptidase N, these studies suggest that the internal NGR sequence in endostatin is constrained and not accessible to bind aminopeptidase N. Furthermore, inhibition of this enzyme may not be directly relevant to the biological activity of endostatin since mouse and human endostatin have different sequences (SGR and NGR, respectively).

Endostatin binds to two distinct classes of proteins on the endothelial cell surface. *α*_5_*β*_1_/*α*_v_*β*_3_ integrins and heparin sulphate-glycosaminoglycan component of the glypican have been reported to be direct targets for endostatin. The domains of endostatin, which are involved in binding the target molecules on endothelial cell surface, have not been completely characterised. The heparin-binding domain of endostatin is composed of a number of positively charged arginine residues (Yamaguchi *et al*, 1999). There are 15 arginine residues in mouse endostatin of which 14 are conserved in human endostatin. In fact, synthetic peptides emcompassing these arginine regions showed antiangiogenic properties (Kasai *et al*, 2002). Other reports showed that mutagenesis of some of the arginine residues either individually or in pairs changed their affinity to heparin. These changes, however, did not affect the biological activity significantly (Sasaki *et al*, 1999; Yamaguchi *et al*, 1999). Our studies show that the P125A-mutation does not affect heparin binding. This conclusion is based on the NaCl concentration required to elute native and mutant protein from heparin affinity column.

However, cell attachment assays showed that P125A-endostatin bound more avidly than native endostatin. Enhanced binding to endothelial cells led to improved biological activity, indicated by the results of endothelial cell proliferation and migration assays. In both the assays, the P125A-mutation increased bioactivity of endostatin. Enhanced binding to endothelial cells also led to improved tumour localisation of endostatin. The homing specificity of endostatin to tumours compared to lung or liver tissue was also improved by P125A-mutation. It is interesting that only tumour accumulation was changed by P125A mutation. These results suggest that the target molecule for P125A is perhaps upregulated in tumour vasculature, thereby facilitating higher binding.

Higher tumour homing also coincided with better *in vivo* activity in matrigel plug assay. Treated matrigels showed tumour cells forming glandular structures. Recently, Hajitou *et al* also reported a similar finding. Endostatin or angiostatin delivered by adenoviral vectors inhibited local invasion and tumour vascularisation of transplanted murine malignant keratinocytes (Hajitou *et al*, 2002).

Real-time PCR data showed that mRNA levels of proangiogenic factors and receptors were downregulated by endostatin treatment, with VEGF and Ang1 downregulated more by P125A-endostatin when compared to native endostatin. Histochemical analysis also showed that VEGF protein levels were decreased in endostatin or P125A-endostatin-treated tumour tissues. Our results are in agreement with those of Calvo *et al* (2002) who showed, in the C3(1)/SV40 transgenic mouse model, that P125A-endostatin treatment suppressed mRNA and protein levels of VEGF, Female C3(1)/SV40 mice treated with P125A-endostatin for a 3-week period showed significant delay in tumour development, reduced tumour burden and increased survival (Calvo *et al*, 2002). Hajitou *et al* (2002) also showed 3–10-fold downregulation of VEGF mRNA expression in endostatin-treated aortic ring. These results indicate that endostatin affects proangiogenic factor expression in the tumour microenvironment.

Slow release of endostatin by alginate encapsulation was used to determine antitumour efficacy. Unlike the bolus injection protocol, alginate-entrapped endostatin was given once a week. This method reduced the cumulative dose to be given to each mouse by seven-fold. Kisker *et al* (2001) also showed that continuous administration using a miniosmotic pump increased the potency of endostatin therapy. Present studies clearly demonstrate that P125A-endostatin inhibits colon carcinoma growth more effectively when compared to the native endostatin.

In summary, these studies show that human endostatin can be genetically modified to improve its ability to bind and inhibit endothelial cells. Higher binding also coincided with changes in potency in inhibiting cell proliferation and migration, and in homing to tumours. Such differences in tumour-homing properties can contribute to improved antitumour activity of mutant endostatin.

The mechanism of enhanced binding to endothelial cells of P125A-endostatin still not entirely clear. It is likely that binding to glypican (supported by heparin-binding data) is not altered by P125A mutation. However, the P125A mutation may expose cryptic determinants in endostatin, which can in turn bind to novel target molecules on endothelial cells. Such an interaction may enhance binding of P125A-endostatin to endothelial cells. We initially hypothesised that aminopeptidase N could be a potential target for P125A-endostatin. However, our current studies clearly demonstrated that P125A-endostatin did not bind to aminopeptidase N. Further work will be necessary to understand the molecular target and mechanism of enhanced binding of P125A-endostatin to endothelial cells.
